# Rhabdomyolysis and exercise-associated hyponatremia in ultra-bikers and ultra-runners

**DOI:** 10.1186/s12970-015-0091-x

**Published:** 2015-06-25

**Authors:** Daniela Chlíbková, Beat Knechtle, Thomas Rosemann, Ivana Tomášková, Jan Novotný, Alena Žákovská, Tomáš Uher

**Affiliations:** Centre of Sports Activities, Brno University of Technology, Brno, Czech Republic; Institute of Primary Care, University of Zurich, Zurich, Switzerland; Faculty of Forestry and Wood Sciences, Czech University of Life Sciences, Prague, Czech Republic; Faculty of Sports Studies, Masaryk University, Brno, Czech Republic; Institute of Experimental Biology, Faculty of Science, Masaryk University, Brno, Czech Republic; Center for Adult Medicine in Bohunice, University Hospital Brno, Brno, Czech Republic

**Keywords:** Mountain biking, Running, Long distances

## Abstract

**Background:**

Exercise-associated hyponatremia (EAH), rhabdomyolysis and renal failure appear to be a unique problem in ultra-endurance racers.

**Methods:**

We investigated the combined occurrence of EAH and rhabdomyolysis in seven different ultra-endurance races and disciplines (*i.e.* multi-stage mountain biking, 24-h mountain biking, 24-h ultra-running and 100-km ultra-running).

**Results:**

Two (15.4 %) ultra-runners (man and woman) from hyponatremic ultra-athletes (*n =* 13) and four (4 %) ultra-runners (four men) from the normonatremic group (*n =* 100) showed rhabdomyolysis following elevated blood creatine kinase (CK) levels > 10,000 U/L without the development of renal failure and the necessity of a medical treatment. Post-race creatine kinase, plasma and urine creatinine significantly increased, while plasma [Na^+^] and creatine clearance decreased in hyponatremic and normonatremic athletes, respectively. The percentage increase of CK was higher in the hyponatremic compared to the normonatremic group (*P* < 0.05). Post-race CK levels were higher in ultra-runners compared to mountain bikers (*P* < 0.01), in faster normonatremic (*P* < 0.05) and older and more experienced hyponatremic ultra-athletes (*P* < 0.05). In all finishers, pre-race plasma [K^+^] was related to post-race CK (*P* < 0.05).

**Conclusions:**

Hyponatremic ultra-athletes tended to develop exercise-induced rhabdomyolysis more frequently than normonatremic ultra-athletes. Ultra-runners tended to develop rhabdomyolysis more frequently than mountain bikers. We found no association between post-race plasma [Na^+^] and CK concentration in both hypo- and normonatremic ultra-athletes.

## Introduction

Exertional rhabdomyolysis, muscle cell breakdown with release of toxic chemicals into plasma and urine, is known as a relatively common response to prolonged strenuous exercise as running [1–21], but less frequently described for cycling [22–25] and triathlon [13]. Acute kidney injury is a potential and serious complication of severe exertional rhabdomyolysis [5, 6, 11, 18, 20, 21, 24, 26–28] and the prognosis is worse if renal failure develops [17–19, 26–29].

Factors proposed for the development of acute renal failure include dehydration secondary to inadequate fluid intake and/or diarrhoea or vomiting [11, 18, 20, 27], rhabdomyolysis [21, 27] and analgesic use [6, 11, 17, 20, 27, 29]. Aggressive drinking can lead to exercise-associated hyponatremia (EAH) and by a currently unknown mechanism induce muscle cell damage which is sufficient to cause kidney failure [6, 9, 14, 29]. According to Noakes [9], any level of overhydration will impair muscle cell function and will be sufficient to lead to a significant muscle cell damage. This can facilitate rhabdomyolysis and myoglobin release, leading to acute renal failure [6, 17, 30]. On the contrary, exertional muscle damage produced by eccentric exercise in healthy individuals can cause creatine kinase elevations without renal impairment [1, 2, 7, 8, 31].

Previous studies related to a proposed link between rhabdomyolysis and EAH [3, 6–8, 14–17, 27, 32–36]. The simultaneous presentation of EAH and rhabdomyolysis creates a complicated and opposing treatment paradox with regard to fluid resuscitation [6, 8, 27]. There are two explanations for the development of EAH and rhabdomyolysis: one causes the other [14, 16, 27, 37] or they occur independently [14]. EAH could promote rhabdomyolysis through changes in intracellular [K^+^] and/or [Ca^++^] concentrations and reduce the stability of the cell membrane, or through direct mechanism decrease cell membrane stability through extracellular fluid osmotically drawn into the muscle cell [6, 14, 27, 35]. If the extracellular [Na^+^] falls during endurance exercise, the intracellular [Ca^++^] is impaired and increases of [Ca^++^] lead to cellular injury, because arginine vasopressin is not able to restore the [Na^+^]/[Ca^++^] balance [14]. During EAH, intracellular [K^+^] is depleted and transferred to the extracellular compartment and myocytes are injured [14]. EAH leads to cell swelling due to extracellular hypoosmolality and their reduction through extrusion of intracellular [K^+^] induces rhabdomyolysis [35]. On the contrary, rhabdomyolysis may cause EAH by arginine vasopressin secretion and fluid leakage into injured muscle [14, 27, 32–35]. Siegel [33] and also Kim *et al*. [12] confirmed the strong relationship between post-exercise interleukin-6 and creatine kinase (CK) concentrations for support that rhabdomyolysis is a stimulus for EAH via the syndrome of inappropriate antidiuretic hormone secretion [33–35].

To prevent and treat rhabdomyolysis, it is necessary to measure CK levels as the most common marker to assess skeletal muscle damage [7, 12] which is also highly correlated to myoglobin [7, 38]. The high CK concentrations could provide an explanation for the high incidence of EAH [36]. The peak CK concentration may be predictive of the development of renal failure [30]. The determination of other muscle enzymes aids less information for a reliable diagnosis [26].

Whether an association exists for the combination of EAH and rhabdomyolysis is currently under debate [6, 27]. Therefore, in the present analysis, we aimed (*i*) to identify athletes who developed simultaneously EAH and rhabdomyolysis. We aimed (*ii*) to expose possible biochemical factors in the hyponatremic and the normonatremic group to better explore this association.

## Methods

### Participants

Institutional review board approval was granted for this study by the local institutional ethics committee. The data were collected from 2012 to 2013 during individual ultra-running and ultra-cycling races. Participants were from the “Czech Championship 24-h mountain bike race” in Jihlava city from 2012 to 2013, the “Bike Race Marathon Rohozec 24 h” in Liberec city in 2012, the “Sri Chinmoy Self-Transcendence Marathon 24-h race” in Kladno city in 2012, the “Trilogy Mountain Bike Stage Race” in Teplice nad Metují in 2012 and 2013 and the 100-km running race in Plzen city in 2013, all held in the Czech Republic.

### Race details

The “Czech Championship 24-h mountain bike race” took place during the second weekend in June 2012 and 2013 at 12:00 on Saturday and finished at 12:00 on Sunday. The course comprised a 9.5 km single-track with an elevation of 220 m. The “Bike Race Marathon Rohozec 24 h” took place on June 9^th^ and finished on June 10^th^ 2012. The course comprised a 12.6 km track with an elevation of 250 m. In both races a single aid station located at the start/finish area was provided by the organizer with a variety of food and beverages. The ultra-mountain bikers were allowed to be supported by additional food and drinks in their pit stops. The “Sri Chinmoy Self-Transcendence Marathon 24-h race” took place from July 21^st^ to July 22^nd^ 2012. The lap was 1 km, situated around an athletic stadium on asphalt with 1 m change in altitude. The organizer provided the athletes with a buffet where both warm and cold food and beverages were offered. The “Trilogy Mountain Bike Stage Race” took place the first week in July 2012 and 2013. The prologue covered 3 km with 300 m difference in elevation. The first stage covered 66 km with 2,200 m of altitude to climb, the second stage was 63 km in length with 2,300 m difference in elevation and the third stage was 78.8 km with 3,593 m. The “Czech championship 100-km running race” was held on July 9^th^ 2013 and the ultra-runners had to run 66 times a lap of 1,500 m in length.

### Procedures, measurements and calculations

At each race, blood and urine measurements were provided during registration the day before each race. Post-race measurements were upon completion of the race. Blood samples were drawn from the seated athlete into heparinized tubes via an antecubital vein. One Sarstedt S-Monovette (plasma gel, 7.5 ml) for chemical analysis was cooled, sent to the laboratory and analysed within 6 h. Blood samples were obtained to determine pre- and post-race plasma [Na^+^], plasma [K^+^], plasma and urine creatinine and plasma CK. Plasma [Na^+^], plasma [K^+^], plasma creatinine and plasma CK were determined using biochemical analyzer Modular SWA P300 (Roche, Basel, Switzerland). Samples of urine were collected in one Sarstedt Monovette for urine (10 ml) and sent to the laboratory. CK was determined in urine samples using biochemical analyzer Modular SWA P300 (Roche, Basel, Switzerland).

### Statistical analysis

Race performance was identical with the absolute ranking in each race due to the connection of runners, mountain bikers and various disciplines (*i.e.* 24-h race, stage race, 100-km race). Continuous data underwent normality test with the D’Agostino and Pearson omnibus normality test. Because data did not pass normality testing, Spearman correlation analyses were used to examine the associations between blood and urinary parameters and pre-race characteristics within the hyponatremic group and the normonatremic group and all finishers. Paired sample *t*-tests or the Wilcoxon signed-rank tests (within different races and within genders, within all finishers, the normonatremic and the hyponatremic group) were used to compare parameters before and after the race as appropriate and to compare continuous measures. Mann–Whitney test was used for group comparisons (hyponatremic and normonatremic, ultra-runners and mountain bikers). Variables as post-race CK and type of race and post-race CK and gender were compared using one-way analysis of variance (ANOVA) and Tukey’s multiple comparison tests. Statistical significance was set at *P* < 0.05.

## Results

Of the 145 ultra-runners and ultra-mountain bikers included in this study, 113 (81.8 %) finishers underwent body mass measurements and provided pre and post-race blood and urine samples. Thirty-two ultra-athletes did not finish their race or they did not undergo all measurements. Thirteen (11.5 %) finishers developed EAH, the remaining subjects (*n =* 100) were normonatremic. Two (1.8 %) ultra-runners (one man and one woman) from all finishers (*n =* 113), that means two (15.4 %) from the hyponatremic ultra-athletes (*n =* 13) and four (3.5 %) from all finishers and four (4 %) ultra-runners (four men) from the normonatremic group (*n =* 100) showed rhabdomyolysis following elevated blood CK levels > 10,000 U/L without the development of renal failure and the necessity of a medical treatment. Data of all cases with rhabdomyolysis (cases 1–6) are presented in Tables 1 and 2. Pre-race variables, age, race performance and body mass of present six cases with rhabdomyolysis including two ones with simultaneously EAH and rhabdomyolysis are shown in Table 1. Percentage changes in their blood and urine parameters (CK, plasma [Na^+^], plasma [K^+^], plasma creatinine, urine creatinine and creatinine clearance) are shown in Table 2.

**Table 1:** Age, body mass, training, pre-race experience, and race performance of subjects with rhabdomyolysis including case 1 and 2 (in bold) with EAH and rhabdomyolysis (*n =* 6)

Case	Age (yrs.)	Sex	Change in body mass (%)	Race (discipline)	Absolute order	Number of finished ultra-marathons (n)	Years as an active biker runner) (yrs.)	Training hours weekly (h)
**1**	**51**	**M**	**−2.9**	**100-km RUN**	**19**	**4**	**27**	**12**
**2**	**38**	**F**	**−2.6**	**24-km RUN**	**1**	**30**	**13**	**15**
3	35	M	−3.4	24-km RUN	2	5	18	10
4	31	M	−0.9	24-km RUN	20	7	5	15
5	26	M	−4.7	24-km RUN	6	2	3	4
6	48	M	−0.2	100-km RUN	2	3	5	11

**Table 2:** Blood and urine parameters subjects with rhabdomyolysis including case 1 and 2 (in bold) with EAH with rhabdomyolysis (*n =* 6)

Case	PreR blood [Na^+^] (mmol/L)	PostR blood [Na^+^] (mmol/L)	Blood [Na^+^] change (%)	Blood [K^+^] change (%)	PreR blood CK U/L	PostR blood CK U/L	Blood CK change (%)	Blood CR change (%)	CRCL change (%)	Urine CR change (%)
**1**	**138**	**134**	**−2.9**	**23.5**	**509.4**	**14512.9**	**2749.0**	**25.0**	**−22.4**	**102.5**
**2**	**137**	**133**	**−2.9**	**−29.2**	**130.0**	**15172.9**	**11571.5**	**14.3**	**−14.7**	**165.6**
3	140	138	−1.4	−25.8	112.9	29702.9	26209.0	11.1	−13.0	143.6
4	138	138	0.0	−40.7	150.6	12786.2	8378.2	10.0	−9.9	212.7
5	141	135	−4.3	−15.3	654.1	18085.9	2665.0	33.3	−28.5	165.0
6	137	141	2.9	36.7	321.9	20280.6	6381.5	50.0	−33.4	485.3

Note: PreR = pre-race, PostR = post-race, [Na^+^] = plasma sodium, [K^+^] = plasma potassium, CK = creatine kinase, CR = creatinine, CRCL = creatinine clearance

We combined the racers by each ultra-endurance disciplines to four categories (SMTB - mountain bike stage racers, 24 MTB - 24-h ultra-mountain bikers, 24 RUN - 24-h ultra-runners and 100RUN - 100-km ultra-runners) (Tables 3, 4, 5 and 6). The limitation was a small number of racers in some races or in genders due to the field nature of research and a toughness of such types of races. We included this fact into our conclusions and mentioned it in the discussion. Nevertheless, we divided racers also by genders to smaller groups to obtain a more accurate characterization of various endurance disciplines by post- minus pre-race differences in body mass, plasma [Na^+^], plasma [K^+^], plasma creatinine, urine creatinine and creatinine clearance (Tables 3, 4, 5 and 6). Post-race CK levels were significantly higher in ultra-runners (*n =* 31) compared to mountain bikers (*n =* 82) (*P* <0.05). There was no difference in post-race plasma [Na^+^] between ultra-runners and mountain bikers (*P* > 0.05). The highest average post-race CK levels were in 24RUNners (7,033 ± 9,360 U/L), follow by 100RUNners (3,211 ± 4,617 U/L), 24MTBers (1,123 ± 1,077 U/L) and SMTBers (918 ± 818 U/L). In 24RUNners post-race increase of CK was significantly higher than in 24MTBers (*r* = 0.40, *P* < 0.01).

**Table 3:** Age, anthropometric, blood and urine characteristics of the stage MTBers (*n =* 32)

	Pre-race	Post- race	Change in absolute	Change in percent	*P*-value
Male multi-stage MTBers (*n =* 28)					
Age (yrs.)	36.7 ± 6.2				
Body mass (kg)	76.6 ± 6.6	75.8 ± 6.3	−0.7 ± 1.5*	−0.9 ± 2.0*	<0.05
Plasma sodium (mmol/L)	142.8 ± 1.9	140.0 ± 2.7	−2.8 ± 2.3**	−1.9 ± 1.6**	<0.01
Plasma potassium(mmol/L)	4.4 ± 0.7	5.2 ± 0.5	0.8 ± 0.9**	21.3 ± 21.7**	<0.01
Plasma creatinine (mg/dL)	0.9 ± 0.1	1.2 ± 0.5	0.3 ± 0.5*	33.3 ± 64.8*	=0.01
Plasma creatine kinase (U/L)	180.7 ± 95.9	1009.4 ± 812.6	828.7 ± 821.2**	586.8 ± 686.9**	<0.01
Plasma creatine clearance (ml/min)	10849.4 ± 1502.9	8953.6 ± 2447.9	−1895.8 ± 2158.7**	−17.4 ± 19.3**	<0.01
Urine creatinine (mmol/L)	7.2 ± 3.9	19.7 ± 17.6	12.5 ± 17.5**	245.8 ± 284.7**	<0.01
Female multi-stage MTBers (*n =* 4)					
Age (yrs.)	39.0 ± 7.2				
Body mass (kg)	65.5 ± 2.8	63.2 ± 2.8	−2.3 ± 1.1*	−3.5 ± 1.6*	<0.05
Plasma sodium (mmol/L)	141.5 ± 2.4	138.8 ± 1.7	−2.8 ± 2.2	−1.9 ± 1.6	0.09
Plasma potassium(mmol/L)	4.7 ± 0.6	4.8 ± 0.4	0.1 ± 0.5	1.8 ± 10.6	0.84
Plasma creatinine (mg/dL)	0.8 ± 0.1	1.0 ± 0.1	0.2 ± 0.1	21.9 ± 15.7	0.68
Plasma creatine kinase (U/L)	118.9 ± 25.4	418.1 ± 413.3	299.2 ± 433.5	308.1 ± 454.3	0.26
Plasma creatine clearance (ml/min)	9862.8 ± 712.2	7943.8 ± 1478.1	−1918.9 ± 1008.3*	−19.8 ± 10.8*	<0.05
Urine creatinine (mmol/L)	7.2 ± 2.6	17.8 ± 1.0	10.6 ± 2.6**	191.7 ± 159.6**	<0.05

Note: Results are presented as mean ± SD; * = *P* < 0.05, ** = *P* < 0.01

**Table 4:** Age, anthropometric, blood and urine characteristics of the 24-hr MTBers (*n =* 50)

	Pre-race	Post- race	Change in absolute	Change in percent	*P*-value
Male 24-hr MTBers (*n =* 38)					
Age (yrs.)	38.4 ± 9.8				
Body mass (kg)	78.8 ± 7.7	77.1 ± 8.0	−1.6 ± 1.5**	−2.0 ± 2.1**	<0.01
Plasma sodium (mmol/L)	138.0 ± 1.6	137.0 ± 2.6	−1.0 ± 2.8*	−0.7 ± 2.0*	<0.05
Plasma potassium(mmol/L)	5.5 ± 1.0	5.0 ± 1.1	−0.5 ± 1.3*	−7.4 ± 23.2*	<0.05
Plasma creatinine (mg/dL)	0.9 ± 0.1	1.2 ± 0.2	0.3 ± 0.2**	32.7 ± 21.8**	<0.01
Plasma creatine kinase (U/L)	207.5 ± 92.3	1316.3 ± 1111.9	1108.9 ± 1099.1**	590.9 ± 560.4**	<0.01
Plasma creatine clearance (ml/min)	10657.2 ± 1614.2	9623.2 ± 1837.4	−1034.1 ± 1121.0*	−9.8 ± 11.7*	<0.05
Urine creatinine (mmol/L)	7.9 ± 3.7	18.4 ± 5.7	10.5 ± 6.5**	212.3 ± 236.4**	<0.01
Female 24-hr MTBers (*n =* 12)					
Age (yrs.)	37.7 ± 7.4				
Body mass (kg)	62.1 ± 5.1	60.0 ± 5.4	−1.9 ± 3.1*	−2.7 ± 5.1*	<0.05
Plasma sodium (mmol/L)	137.5 ± 2.5	136.6 ± 2.0	−0.9 ± 3.0	−0.6 ± 2.2	0.31
Plasma potassium(mmol/L)	5.1 ± 0.8	4.7 ± 0.6	−0.5 ± 0.7*	−8.2 ± 12.0*	<0.05
Plasma creatinine (mg/dL)	0.9 ± 0.1	1.0 ± 0.1	0.1 ± 0.2	13.4 ± 20.0	0.05
Plasma creatine kinase (U/L)	148.7 ± 75.7	568.9 ± 293.4	420.2 ± 285.3**	349.9 ± 343.7**	<0.01
Plasma creatine clearance (ml/min)	9365.7 ± 1579.9	8228.8 ± 2228.7	−13.0 ± 17.3	−12.5 ± 15.7	0.05
Urine creatinine (mmol/L)	5.4 ± 2.4	15.7 ± 4.9	10.3 ± 4.4**	252.4 ± 207.8**	<0.01

Note: Results are presented as mean ± SD; * = *P* < 0.05, ** = *P* < 0.01

**Table 5:** Age, anthropometric, blood and urine characteristics of the 24-hr ultra-runners (*n =* 12)

	Pre-race	Post- race	Change in absolute	Change in percent	*P*-value
Male 24-hr ultra-runners (*n =* 8)					
Age (yrs.)	37.5 ± 8.1				
Body mass (kg)	71.1 ± 5.9	70.0 ± 6.7	−1.1 ± 1.2*	−1.6 ± 1.8*	<0.05
Plasma sodium (mmol/L)	140.1 ± 1.2	139.1 ± 2.0	−1.0 ± 2.3	−0.7 ± 1.7	0.26
Plasma potassium(mmol/L)	6.7 ± 1.1	5.1 ± 0.6	−1.6 ± 1.3*	−21.8 ± 17.4*	<0.05
Plasma creatinine (mg/dL)	0.9 ± 0.1	0.9 ± 0.2	0.0 ± 0.1	10.6 ± 12.7	0.06
Plasma creatine kinase (U/L)	224.0 ± 192.0	8813.8 ± 10536.4	8589.8 ± 10500.7*	5782.1 ± 8659.2*	<0.05
Plasma creatine clearance (ml/min)	10657.2 ± 1614.2	9623.2 ± 1837.4	−1034.1 ± 1121.0*	−9.8 ± 11.7*	<0.05
Urine creatinine (mmol/L)	7.5 ± 1.3	17.5 ± 2.6	10.0 ± 2.7**	139.2 ± 47.9**	<0.01
Female 24-hr ultra-runners (*n =* 4)					
Age (yrs.)	40.0 ± 7.7				
Body mass (kg)	56.9 ± 5.2	56.3 ± 5.1	−0.6 ± 0.6	−1.0 ± 1.2	0.16
Plasma sodium (mmol/L)	139.8 ± 2.6	137.8 ± 3.4	−2.0 ± 3.4	−1.4 ± 2.4	0.32
Plasma potassium(mmol/L)	6.2 ± 0.3	4.6 ± 0.2	−1.7 ± 0.2**	−26.4 ± 2.9**	<0.01
Plasma creatinine (mg/dL)	0.7 ± 0.1	0.7 ± 0.1	0.01 ± 0.0	9.0 ± 7.3	0.18
Plasma creatine kinase (U/L)	99.0 ± 23.5	5507.1 ± 6600.4	5408.1 ± 6580.2**	4765.0 ± 4806.5**	0.19
Plasma creatine clearance (ml/min)	10353.4 ± 795.3	9547.0 ± 845.9	−806.4 ± 838.7	−7.6 ± 7.9	0.15
Urine creatinine (mmol/L)	4.5 ± 2.1	15.2 ± 0.2	10.7 ± 1.9**	327.8 ± 276.6**	<0.01

Note: Results are presented as mean ± SD; * = *P* < 0.05, ** = *P* < 0.01

**Table 6:** Age, anthropometric, blood and urine characteristics of the 100-km ultra-runners (*n =* 19)

	Pre-race	Post- race	Change in absolute	Change in percent	*P*-value
Male 100 km ultra-runners (*n =* 4)					
Age (yrs.)	43.9 ± 9.6				
Body mass (kg)	67.0 ± 8.5	65.1 ± 7.8	−1.8 ± 1.3**	−2.6 ± 1.7**	<0.01
Plasma sodium (mmol/L)	139.6 ± 2.2	137.8 ± 2.9	−1.8 ± 3.6	−1.3 ± 2.6	0.09
Plasma potassium(mmol/L)	6.2 ± 1.0	5.6 ± 0.7	−0.6 ± 1.6	−6.2 ± 24.9	0.18
Plasma creatinine (mg/dL)	0.9 ± 0.2	1.1 ± 0.3	0.2 ± 0.3	25.8 ± 48.1	0.05
Plasma creatine kinase (U/L)	227.1 ± 148.9	4357.1 ± 5822.5	4130.0 ± 5742.7*	1925.5 ± 2180.3*	<0.05
Plasma creatine clearance (ml/min)	8985.3 ± 2219.0	7327.7 ± 2080.2	−1657.5 ± 2146.8**	−16.7 ± 17.9*	<0.05
Urine creatinine (mmol/L)	6.6 ± 4.6	18.5 ± 4.1	11.9 ± 5.8**	365.2 ± 332.1**	<0.01
Female 100 km ultra-runners (*n =* 5)					
Age (yrs.)	40.6 ± 5.8				
Body mass (kg)	59.0 ± 7.7	57.6 ± 6.6	−1.4 ± 1.2	−2.3 ± 1.6	0.05
Plasma sodium (mmol/L)	138.4 ± 2.3	136.0 ± 1:6	−2.4 ± 2.7	−1.7 ± 1.9	0.12
Plasma potassium(mmol/L)	5.2 ± 0.9	5.5 ± 0.9	0.3 ± 0.6	7.3 ± 13.0	0.28
Plasma creatinine (mg/dL)	0.8 ± 0.1	1.0 ± 0.2	0.2 ± 0.1*	25.6 ± 20.6*	<0.05
Plasma creatine kinase (U/L)	251.1 ± 187.7	1949.3 ± 1357.3	1698.2 ± 1413.2*	990.7 ± 748.9*	0.06
Plasma creatine clearance (ml/min)	9335.1 ± 1748.4	7463.4 ± 2240.3	−1871.7 ± 1199.4*	−20.6 ± 12.9*	<0.05
Urine creatinine (mmol/L)	8.2 ± 4.8	19.4 ± 7.4	11.2 ± 6.7*	192.2 ± 153.0*	<0.05

Note: Results are presented as mean ± SD; * = *P* < 0.05, ** = *P* < 0.01

**Fig. 1:**
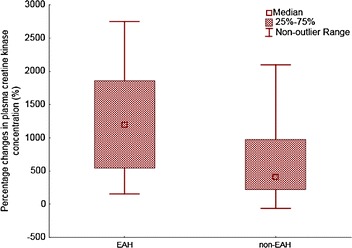
The percentage increase of CK and decrease in creatine clearance were higher in the hyponatremic compare to the normonatremic group (*P* < 0.05)

**Fig. 2:**
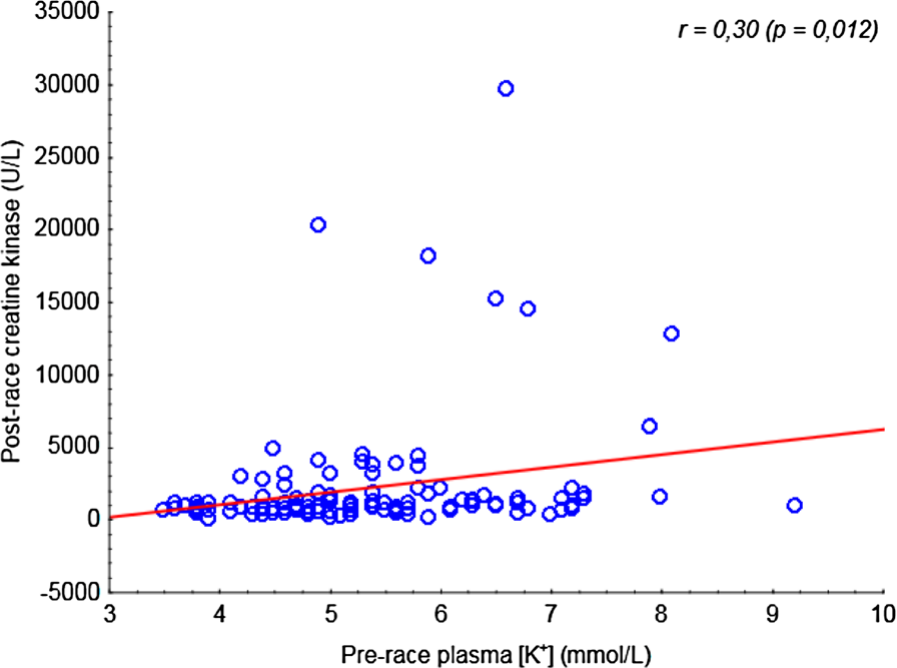
Pre-race plasma [K^+^] related to post-race CK in all finishers (*P* < 0.05)

We compared the hyponatremic (*n =* 13) and the normonatremic (*n =* 100) group of ultra-athletes. An average race performance (absolute order in each race) was 38.5 ± 47.7 in the hyponatremic group and 49.5 ± 43.3 in the normonatremic group without significant difference between both groups. Race performance correlated negatively to post-race (*r* = −0.37, *P* <0.05) and percentage change (*r* = −0.31, *P* < 0.05) in CK only in the normonatremic group. An average age of hyponatremic athletes was 39.5 ± 6.9 years, normonatremic athletes 38.6 ± 8.5 years. Age was associated with post-race (*r* = 0.58, *P* < 0.05) and percentage change (*r* = 0.61, *P* < 0.05) in CK in the hyponatremic group. The number of years as an active runner (biker) was related to the percentage change (*r* = 0.75, *P* < 0.05) in CK only in the hyponatremic group. Neither the number of finished ultra-marathons nor the total number of hours spent by training weekly, nor gender was related to post-race CK concentration (*P* > 0.05) in both groups. In the hyponatremic group, CK increased by 3,456 ± 4,982 U/L and 1.9 ± 2.9 %, respectively, from baseline (*P* < 0.01). In the normonatremic group CK increased by 1,831 ± 4,073 U/L and 1.1 ± 2.8 %, respectively, from baseline (*P* < 0.01). The percentage increase of CK was higher in the hyponatremic group (*P* < 0.05) (Fig. 1). Post-race CK was related to pre-race plasma [K^+^] in all finishers (*P* < 0.05) (Fig. 2). On the contrary, post-race or percentage change in CK was not associated with percentage change in body mass, post-race plasma [Na^+^], plasma [K^+^], plasma and urine creatinine and creatine clearance in all finishers, hyponatremic or normonatremic ultra-athletes (*P* > 0.05).

In the hyponatremic group, post-race plasma [Na^+^] decreased by 5.8 ± 2.1 mmol/L (4.1 ± 1.5 %) (*P* < 0.01). In the normonatremic group, post-race plasma [Na^+^] decreased by 1.2 ± 2.4 mmol/L, (0.8 ± 1.7 %) (*P* < 0.01). Percentage change in plasma [Na^+^] was significantly higher in hyponatremic ultra-athletes (*P* < 0.05). Plasma [K^+^] remained stable post-race (*P* > 0.05) in hyponatremic ultra-athletes. Post-race plasma [K^+^] decreased by 5.1 ± 0.9 mmol/L post-race, (1.3 ± 25.3 %) (*P* < 0.05) in normonatremic ultra-athletes. Pre-race plasma [K^+^] was not related to post-race plasma [K^+^] in all finishers, hyponatremic or normonatremic ultra-athletes (*P* > 0.05). Post-race plasma creatinine increased by 1.1 ± 0.5 mg/dL (39.5 ± 71.0 %) (*P* < 0.05) in hyponatremic finishers. Post-race plasma creatinine increased by 1.1 ± 0.3 mg/dL (25.2 ± 34.1 %) (*P* < 0.01) in normonatremic finishers. Post-race urine creatinine increased by 7.9 ± 7.0 mmol/L (216 ± 287 %) (*P* < 0.01) in the hyponatremic group. Post-race urine creatinine increased by 9.5 ± 5.7 mmol/L (229 ± 235 %) (*p* < 0.01) in the normonatremic group. Post-race plasma creatine clearance decreased by 2,454 ± 2,428 ml/min (21.7 ± 18.4 %) (*P* < 0.01) in the hyponatremic group. Plasma creatine clearance decreased by 1,913 ± 1,815 ml/min (18.0 ± 15.6 %) (*P* < 0.01) in the normonatremic group. In comparison of the normonatremic and the hyponatremic group percentage change in plasma [K^+^], plasma and urine creatinine and creatine clearance did not differ significantly between both groups.

## Discussion

The most important result was that two (15.4 %) ultra-runners from 13 hyponatremic and four (4.0 %) ultra-runners from 100 normonatremic finishers (5.3 % from a total number of finishers) developed post-race CK levels higher than 10,000 U/L without the occurrence of renal failure and the necessity of a medical treatment.

### Pre-race characteristics of cases with rhabdomyolysis (cases 1–6)

The present case 1 (51-year-old man) and case 2 (38-year-old woman) with EAH and rhabdomyolysis were among the faster, but not younger finishers in their races. Female 24RUNner case 2 was even the first according to the absolute order among both genders. The 153-km ultra-runners with EAH and rhabdomyolysis (three men and one woman) exhibited an average age of 38 ± 8 years in a study by Ellis *et al*. [14]. In a recent study published by Hoffman *et al.* [8], a 161-km hyponatremic ultra-runner with rhabdomyolysis was 53 years old. In the study of Boulter *et al.* [17], three (from four with acute renal failure) hyponatremic male 89-km ultra-marathoners noted their average age of 34.0 ± 8.7 years. In the study of Bruso *et al*. [6], 161-km ultra-runners with EAH and rhabdomyolysis were men with an average age of 39 ± 7 years and they tended to be younger and faster than those not developing EAH with rhabdomyolysis. The present normonatremic male cases with rhabdomyolysis (cases 3–6) with mean age 35.0 ± 9.4 were among the faster finishers and they were on average younger compared to the rest of the normonatremic athletes. As described by Hoffman *et al*. [7] in their study of 161-km finishers of “Western States Endurance Run” (WSER) with rhabdomyolysis, blood CK concentrations were not related to finish time, age or the number of prior similar completed races. The comparison of the present hyponatremic cases (1,2) and normonatremic cases (3–6) with rhabdomyolysis was impossible due to the low number of cases. Nevertheless, when we compared all hyponatremic and normonatremic cases, the present older and more trained (*i.e.* more years spent by running/biking) hyponatremic ultra-athletes developed higher post-race CK concentrations than the younger ones and the less trained hyponatremic ultra-athletes. Moreover, the present faster normonatremic finishers developed higher post-race CK concentrations than the slower normonatremic finishers. On the contrary, race experience (*i.e.* the number of finished ultra-marathons) or the training frequency and length (*i.e.* number of training hours per week) related to an increased CK neither in hyponatremic, nor in normonatremic ultra-athletes. Gender was not related to CK in the present ultra-athletes. The rare incidence of women with EAH and rhabdomyolysis probably reflects the ratio of female to male finishers in similar ultra-endurance races [6].

### Creatine kinase concentrations

Normal CK post-race values are up to ~ 2,000 U/L [9]. In accordance to Sinert *et al*. [31], inclusion criteria were an elevated CK of more than 500 U/L and they reported exertional rhabdomyolysis with admission CK levels between 700 U/L and 167,000 U/L. CK levels of roughly 500–1500 U/L [24] or approximately over 2,000 U/L are used as the criterion for statin myopathy [39, 40] and CK above 10,000 U/L as diagnosis of rhabdomyolysis [27, 31]. In the present study, CK ≥ 2,000 U/L and associated myopathy developed three (23.1 %) hyponatremic (one male and one female 100RUNners and one male 24MTBer) and fifteen (15 %) normonatremic (six male 24MTBers, two male and one female 24RUNners, three male and one female 100RUNners and two male SMTBers) ultra-athletes. Overall, eighteen (15.9 %) of all present ultra-athletes (*n =* 113) developed exercise-associated myopathy.

Cases 1 and 2 developed biochemical EAH and exercise-induced rhabdomyolysis with CK levels of 14,512 U/L and 15,172 U/L, respectively. The rest of the hyponatremic group (*n =* 11) had CK range from 691 U/L to 3,163 U/L. In the normonatremic cases 3, 5 and 6 with rhabdomyolysis post-race CK increased in the range from 12,768 U/L to 20,280 U/L. Aside from cases 1 and 2, normonatremic cases 3, 5 and 6 exhibited CK concentrations higher than 14,512 U/L, the lower initial post-race value of two cases with EAH and rhabdomyolysis. The study of the 153-km ultra-runners with EAH and rhabdomyolysis described CK levels in the range from 15,636 U/L to more than 100,000 U/L [14]. Rhabdomyolysis in combination with EAH in one male athlete was presented by Putterman [35] with CK 1,545 U/L which peaked at 10,300 U/L. Boulter *et al.* [17] described in three 89-km runners with EAH and rhabdomyolysis CK levels in a wide range from 5,718 U/L to 48,934 U/L. Hoffman *et al.* [16] observed higher blood CK concentrations among those with EAH than those not developing EAH at the 2011 WSER. In 161-km ultra-run race mean CK concentrations were even 54,583 U/L in the hyponatremic group and 30,335 U/L in the normonatremic group [16]. Percentage change in CK was significantly higher in the present hyponatremic compare to normonatremic ultra-athletes. Hyponatremic finishers with an average post-race CK 3,658 ± 5,029 U/L tended to develop exercise induced rhabdomyolysis more than present normonatremic ultra-athletes with an average post-race CK 2,025 ± 4,094 U/L.

We could not compare validly different kinds of races and ultra-disciplines due to the small number of participants in some observed races. Nevertheless, post-race plasma CK significantly increased in male and female finishers in all races (SMTB, 24MTB, 24RUN and 100RUN), except female SMTBers with a non-significant increase. Notwithstanding, in comparison of all ultra-runners (*n =* 31) and all mountain bikers (*n =* 82) post-race CK levels were significantly higher in the present ultra-runners. Despite a dissimilar number of participants in each race discipline we found the highest post-race CK levels in the 24RUNners and the 100RUNners with a significantly higher increase of post-race CK in 24RUNners compared to 24MTBers. Moreover, cases 1 and 6 were from 100RUN and cases 2, 3, 4 and 5 were 24RUNners. In accordance to Hoffman *et al*. [8] mild to moderate elevations of CK are common in long running distance and exertional rhabdomyolysis is often associated with EAH. Skenderi *et al*. [1] assumed that prolonged exercise at even moderate intensity can induce asymptomatic exertional rhabdomyolysis. In their study of 246-km ultra-runners an increase of post-race CK was 43,763 U/L; nevertheless, the ultra-runners did not require hospitalisation. The reasons for an increase in CK could be also the duration of races and the large eccentric component of ultra-running races [7, 12]. On the contrary, a low increase of CK of 542 U/L during 24 h after the exercise appeared after two hours of cycling [25]. The groups of ultra-runners and MTBers were not equal; nevertheless, the present ultra-runners tended to develop more frequently exercise-induced rhabdomyolysis than the present ultra-MTBers.

Normonatremic cases 3 and 6 (1.8 % from the total of 113 ultra-athletes) developed post-race CK concentration of 20,280 U/L, a level associated with renal failure [31]. Acute kidney injury is a complication of severe rhabdomyolysis (CK > 60,000 U/L to 80,000 U/L) and the prognosis is worse with renal failure [38]. However, no present finisher developed acute kidney injury or renal failure or need a medical treatment. In the present study the post-race CK levels were not as high as in other studies. However, the average increase was from 2,665 % to 26,209 % in all cases with rhabdomyolysis. Factors associated with acute renal failure include rhabdomyolysis with CK concentrations higher than 20,000 U/L or higher than five times the normal value [31, 38]. However, no defined level exists. Following Meijer *et al.* [30], the risk of acute renal injury in rhabdomyolysis is low at CK levels lower than 15,000 U/L to 20,000 U/L. Acute renal injury with CK levels at 5,000 U/L usually occurs with hypovolemia (low circulating volume) or aciduria (acidic urine) [26, 27, 29, 31]. However, exercise may induces factors protect against hypovolemia and aciduria. Exercise-induced rhabdomyolysis with mean CK concentrations up to 40,000 U/L [1, 31] has not been reliable to diagnose renal failure [17]. Blood CK concentration was reported from the 161-km WSER in 1980 through 1983 [3, 4], 1995 [5], 2009 [6] and 2010 [7] and Bruso *et al*. [6] first defined the relationship between EAH and rhabdomyolysis in the 161-km ultra-run in five runners with CK values of 40,000 U/L. CK concentrations in the 2010 WSER finishers were higher than values previously reported [7]. Hoffman *et al*. [7] suggested that stress caused by trail running race with its challenging conditions produced severe muscle damage. However, CK values higher than 20,000 U/L are common for this type of event and seldom result in detrimental consequences. Hoffman *et al.* [7] found no athlete with acute renal failure, despite high mean CK levels of 32,956 U/L. Thirty-nine ultra-runners finishing a 245-km race with CK values exceeding 40,000 U/L also had not been shown to have acute renal failure [1]. On the contrary, four cases of acute renal failure in the Comrades marathon had various levels of CK values of 39,000 U/L, 29, 800 U/L, 24,120 U/L and 2,220 U/L [17]. In the cohort of twenty-six patients with severe rhabdomyolysis the average level of CK with 38,351 U/L was predicted the development of acute renal failure [30]. Nevertheless, exercise-induced rhabdomyolysis rarely progresses to acute renal failure [27, 31] and less severe forms of rhabdomyolysis or in cases of hyperCKemia (*i.e.* chronic or intermittent muscle destruction) present with no renal failure [26]. Moreover, exertional muscle damage produced by eccentric exercise can lead to an elevated CK without renal impairment [2, 39]. CK level is, therefore, not useful in distinguishing acute renal failure [2, 31, 41]. Given the wide range of CK levels the value of CK is limited to diagnose rhabdomyolysis [30]. Factors for renal failure in cases of exertional rhabdomyolysis in marathon running could be a pre-existing viral/bacterial infection, heat stress, dehydration, latent myopathy, NSAID (non-steroidal anti-inflammatory drugs), other drugs or analgesic use [2, 27, 31]. In the present study we were not able to observe these factors in ultra-athletes. However, the present results support hypothesis that the magnitude of elevated CK do not have exactly and always predict acute renal failure [2, 31, 38, 39, 41].

### Plasma [Na^+^] and [K^+^] concentrations

Plasma [Na^+^] decreased in cases 1 and 2 with rhabdomyolysis and EAH and cases 3 and 5 with rhabdomyolysis within the hyponatremic and the normonatremic group with a significantly higher increase in the hyponatremic group. In different kinds of the present races limited by various numbers of participant’s plasma [Na^+^] decreased in all ultra-disciplines. Hyponatremia as the most common electrolyte disorder associated with ultra-running and muscle-cell swelling with mechanical stress caused by running (footrace) may result in skeletal muscle damage, rhabdomyolysis, or acute renal injury [27]. However, we found no study about the occurrence of rhabdomyolysis and EAH in cycling races. Also the present post-race CK levels were significantly higher in the ultra-runners and no mountain biker presented EAH with rhabdomyolysis or just rhabdomyolysis. Blood [Na^+^] and CK concentrations were negatively correlated in 161-km ultra-runners [16]. In all present finishers (*n =* 113), in hyponatremic and in normonatremic finishers, CK concentrations were not associated with plasma [Na^+^]. In a recent study of Hoffman and Stuempfle [15] from the WSER in 2011 and 2013, a significant relationship between plasma [Na^+^] and CK concentration was also evident, however, without the difference between the hyponatremic and the normonatremic group due to wide variability in creatine kinase concentrations. Similarly, in the study of Hoffman *et al.* [7], the relationship between blood CK and [Na^+^] did not reach statistical significance.

The recognition of electrolyte abnormalities associated with rhabdomyolysis and induced acute kidney injury like hyperkalemia (serum [K^+^] ≥ 5.5 mmol/L) is important to remove [K^+^] from the body [26]. Hyperkalemia can be classified according to serum [K^+^] into mild (5.5 - 6.5 mmol/L), moderate (6.5 - 7.5 mmol/L) and severe (>7.5 mmol/L) hyperkalemia [42]. A metabolic disorder known to cause EAH and rhabdomyolysis is also hypokalemia, when [K^+^] depletion due to cell swelling eventually induces rhabdomyolysis [35]. None of present ultra-athletes showed post-race hypokalemia. Present case 1 developed post-race plasma [K^+^] 5.2 mmol/L and case 2 plasma [K^+^] of 4.6 mmol/L. A study of four athletes with EAH and rhabdomyolysis showed plasma [K^+^] in the range from 4.1 mmol/L to 4.9 mmol/L [14]. The present levels of post-race plasma [K^+^] were higher than in the study of Bruso *et al*. [6] with an average of 4.0 mmol/L (range 3.4 - 4.9 mmol/L) in his 5 cases with EAH and rhabdomyolysis and higher than in the study of Boulter *et al.* [17] with an average of 4.4 mmol/L.

The interesting finding was that the present cases 1 and 2 showed pre-race values of plasma [K^+^] (6.8 mmol/L and 6.5 mmol/L, respectively) which tended to be higher than in other hyponatremic ultra-athletes. In the present cases with just rhabdomyolysis (cases 3–5), post-race plasma [K^+^] ranged from 4.8 mmol/L to 5.0 mmol/L, only case 6 reached 6.7 mmol/L (moderate post-race hyperkalemia). Another finding was the post-race decrease of plasma [K^+^] in all cases (1–5), except case 6. The reason could be probably pre-race mild to severe hyperkalemia in cases 1–5 due to the range of pre-race plasma [K^+^] from 5.9 mmol/L to 8.1 mmol/L (case 4 with 8.1 mmol/L). On the contrary, case 6 presented with a pre-race level of 4.9 mmol/L and therefore probably exhibited post-race increase of plasma [K^+^]. The average post-race plasma [K^+^] in the present normonatremic finishers was in the range from 3.8 mmol/L to 8.2 mmol/L and twenty-seven (27 %) athletes developed levels higher than 5.5 mmol/L. Their average pre-race plasma [K^+^] was 5.4 ± 1.2 mmol/L (range 3.5 mmol/L to 9.2 mmol/L) with fifteen (15 %) ultra-athletes with pre-race level ≥ 5.5 mmol/L. Hyponatremic finishers had an average post-race plasma [K^+^] in the range from 4.4 mmol/L to 6.5 mmol/L with four (30.8 %) finishers with a level higher than 5.5 mmol/L. Even in five (38.5 %) hyponatremic finishers pre-race plasma [K^+^] reached levels of ≥ 5.5 mmol/L. Post-race plasma [K^+^] significantly decreased in both genders in 24MTBers and 24RUNners and non-significantly in male 100RUNners despite the limitation of different number of finishers in each ultra-endurance discipline.

Another finding was that pre-race plasma [K^+^] showed an average value of 6.7 mmol/L and 6.2 mmol/L in male and female 24RUNners, respectively, 5.5 mmol/L and 6.2 mmol/L in male 24MTBers and male 100RUNners, respectively. On the contrary, the lowest pre-race level was 4.4 mmol/L in male SMTBers, 4.7 mmol/L in female SMTBers, 5.2 mmol/L in female 100RUNners, and we simultaneously found a significant increase in post-race plasma [K^+^]. Overall, despite the genders and the various numbers of racers in each ultra-disciplines 57.9 % 24RUNners, 42.0 % of 24MTBers, 16.7 % 100RUNners and 6.3 % of SMTBers showed pre-race hyperkalemia and 47.4 % 100RUNners, 28.1 % SMTBers, 20 % 24MTBers and 16.7 % 24RUNners developed post-race hyperkalemia.

A further interesting finding was that we found an association between pre-race levels of plasma [K^+^] and post-race CK levels in the present finishers. The ultra-athletes with higher pre-race plasma [K^+^] developed post-race higher CK levels. It also appears that races with a higher occurrence of pre-race hyperkalemia tended to a lower occurrence of hyperkalemia post-race and conversely. However, there was no significant relationship between pre-race plasma [K^+^] and post-race plasma [K^+^] considering all present ultra-athletes. The presence of mild pre-race or post-race hyperkalemia in some present hyponatremic and normonatremic cases supports either excessive [K^+^] ingestion pre-race or during the race, ingestion of NSAID, reduced renal excretion or tissue breakdown as in rhabdomyolysis [42]. Notwithstanding, we found no association between post-race plasma [K^+^] and CK concentration. We have to take into account that some results could be also caused by pseudohyperkalemia from leakage of [K^+^] from the intracellular space during or after blood sampling in field conditions [42].

### Plasma and urine creatinine concentrations

Muscle injury releases creatine and increases blood creatinine as one of parameters of myocellular damage [31, 39]. Biochemical criteria for acute renal injury mean a blood creatinine concentration more than 2.0 mg/dL and 1.5 times of the estimated baseline [27, 31, 39]. Post-race plasma and urine creatinine significantly increased and creatine clearance decreased in all cases with rhabdomyolysis (cases 1–6), in the hyponatremic and the normonatremic group and in both genders in all present different races. Current cases 1 and 2 with EAH and rhabdomyolysis developed an average post-race creatinine level of 0.95 mg/dL (1.0 mg/dL and 0.8 mg/dL). In accordance to the study of Bruso *et al*. [6], their average level of post-race blood creatinine in cases with EAH and rhabdomyolysis was higher than in the present study. Three cases in the study of Bruso *et al*. [6] developed acute renal failure with higher blood creatinine (2.8 mg/dL to 4.9 mg/dL) than two cases without renal failure (1.1 mg/dL to 1.2 mg/dL), however, without a difference in CK concentrations. Cases 3–6 with just rhabdomyolysis showed an average post-race creatinine level in the range from 1.0 mg/dL to 1.2 mg/dL. In the study of Hoffman *et al*. [38], the range for blood creatinine levels was similar as in the present cases with rhabdomyolysis in the range from 1.1 mg/dL to 1.4 mg/dL. The present hyponatremic group developed post-race creatinine concentration in the range from 0.8 mg/dL to 2.6 mg/dL and the normonatremic group from 0.7 mg/dL to 3.4 mg/dL; however, without a significant difference between both groups.

Present creatinine levels above the upper limit of normal were only found in one hyponatremic male SMTBer (2.6 mg/dL, plus 271.4 %) and one male normonatremic SMTBer (3.4 mg/dL, plus 240.0 %) from a total of 113 ultra-athletes. The 42-year old hyponatremic male SMTBer (post-race plasma [Na^+^] 134 mmol/L) developed also high pre- and post-race levels of plasma [K^+^] (5.5 and 5.7 mmol/L), minus 3.8 percentage change in body mass; the highest percentage decrease (minus 74.1 %) of creatine clearance within all finishers; however with post-race CK just 1,197 U/L. The 26-year old normonatremic SMTBer showed plasma [Na^+^] 140 mmol/L, pre- and post-race levels of plasma [K^+^] 3.6 and 4.9 mmol/L, plus 0.6 % percentage change in body mass, the second highest percentage decrease (minus 70. 4 %) of creatine clearance within all finishers; however similarly with post-race CK only 1,168 U/L. Both current cases presented post-race without the development of renal failure and the necessity of a medical treatment. Neumayr *et al*. investigated the effect of marathon cycling on renal function in recreational and professional road cyclists [22, 23] with no evidence for a significant skeletal muscle damage and a reduced renal perfusion responsible for the slight impairment of renal function after marathon cycling [22]. In a study of the 38 recreational male marathon cyclists the increases in plasma creatinine were 20 %, the decrease of creatinine clearance was similar 18 % [22]. In 16 professional road cyclists (525-km race), plasma creatinine rose by 33 %, the decrease of creatine clearance was 25 % [23]. Two multi-stage mountain bikers in the present study developed the highest levels of post-race plasma [K^+^] and the lowest concentrations of creatine clearance; however, both with low post-race CK concentrations. Moreover, these hyponatremic multi-stage bikers showed a higher percentage increase in post-race CK levels than normonatremic biker. In the study of Hoffman *et al.* [41], 4 % of their ultra-runners met the criteria for injury (*i.e.* blood creatinine 2.0 times of the estimated baseline) and 29 % for risk (*i.e.* blood creatinine 1.5 times of the estimated baseline) of acute renal injury and those meeting the injury criteria had higher CK concentrations. Nevertheless, very few runners seek or require medical treatment for acute renal injury [41]. In all present SMTBers plasma creatinine rose by 33.3 % ± 64.8 in men and by 21.9 ± 15.7 % in women with minus 17.4 ± 19.3 % change in male creatine clearance and minus 19.8 ± 10.8 % change in female creatine clearance and their post-race creatine levels and post-race percentage changes in CK were the lowest from all different ultra-disciplines in the present study. These data confirm that the strains of ultramarathon cycling regardless these two presented cases did not influence their renal function. Even though, Khalil *et al.* [43] suggested that acute renal failure is defined with serum creatinine >3-fold from baseline, or > 4 mg/dL with an acute rise of 0.5 mg/d L or greater. The present multi-stage mountain bikers fulfilled these conditions; they did not seek or required medical treatment for acute renal injury. In the present study, correlations between post-race CK values and plasma creatinine, urine creatinine or creatine clearance values were not found.

### Limitations

We have to take into account that the number of present ultra-athletes in various disciplines and gender representation was different. Further we did not observe a urine dipstick for a urine colour, blood and myoglobin. However, direct measurement of myoglobin is less reliable and not clinically useful [44]. Moreover, increased CK levels are associated with increased blood myoglobin [7, 38]. Hereafter, a development of rhabdomyolysis can delay several hours after the occurring of EAH due to the changes in intracellular [K^+^] and the restoring of cellular volume [14]. Peak CK level is about 48 to 96 h after its presentation [44]. In the marathon cyclists renal parameters remained elevated during 24 h of recovery [22, 23]. We had no possibility to observe ultra-athletes one or more days after the race; otherwise the danger of rhabdomyolysis could be higher.

### Practical applications

The value of CK as a prognostic tool is limited, due to the wide inter-individual range of CK levels. In situations where the diagnosis of EAH is uncertain, fluid restriction during or after the race is contraindicated in case of dehydration and rhabdomyolysis with impending acute kidney injury [8, 27, 29, 41].

## Conclusions

The present hyponatremic ultra-athletes tended to develop exercise-induced rhabdomyolysis more than normonatremic ultra-athletes. Ultra-runners tended to develop rhabdomyolysis more than mountain bikers. The present work does not clarify the mechanism that might be involved in the suggested link between rhabdomyolysis and EAH.

### Consent

Written informed consent was obtained from all testing subjects for the publication of this report and any accompanying images.
